# Stereoscopic depth without monocular recognition

**DOI:** 10.1177/20416695251329309

**Published:** 2025-04-24

**Authors:** Nicholas J. Wade

**Affiliations:** University of Dundee, Dundee, UK

**Keywords:** anaglyphs, random-dot stereograms, binocular vision, Wheatstone, Cajal, Julesz

## Abstract

The search for stereograms that reveal depth features to two eyes that are concealed from each alone commenced with announcement of the invention of the stereoscope by Wheatstone in 1838. The paired figures he presented to the eyes were mostly simple outline drawings of geometrical objects, in order to reduce or remove monocular indications of depth. One stereogram, consisting of dots, yielded depth without monocular recognition; later others did so with more complex stereograms. Most notably in 1960, Julesz achieved this with computer-generated random-dot stereograms. Prior to Julesz similar patterns were hand-made, photographed and paired to yield stereograms by Cajal, Mobbs, Kompaneysky, and Aschenbrenner. Wheatstone did not recognise the significance of his simple dot stereogram possibly because he was interested in representing objects rather than surfaces stereoscopically. Thus, it can be argued that the precursors of random-dot stereograms were produced by Wheatstone in his article describing the invention of the stereoscope.

## How to cite this article

Wade, N. J. (2025). Stereoscopic depth without monocular recognition. *i-Perception, 16*(0), 1–16. https://doi.org/10.1177/20416695251329309

## Introduction

Charles Wheatstone (1838) demonstrated stereoscopic depth perception with the aid of his invention, the stereoscope. The paired figures he presented to the eyes were simple drawings, mostly of geometrical objects

For the purposes of illustration I have employed only outline figures; for had either shading or colouring been introduced it might be supposed that the effect was wholly or in part due to these circumstances, whereas by leaving them out of consideration no room is left to doubt that the entire effect of relief is owing to the simultaneous perception of the two monocular projections, one on each retina. (1838, p. 376)

That is, Wheatstone wished to avoid the use of monocular aids for conveying depth or distance so that only disparity was operating. This was not possible with his outline figures but one stereogram, consisting of lines of dots, yielded depth without monocular recognition. The dot stereogram was devised to demonstrate that monocular rows of dots with differing separations in each eye could be combined binocularly; they were also seen in depth. The significance of this stereogram was not appreciated by Wheatstone nor by later streoscopists. Although others did make more complex stereograms that yielded depth without monocular recognition, we associate this with the random-dot stereogram (RDS) devised by Béla Julesz (1960, 1971) who used a computer to produce them. He appreciated the novelty of the technique as well as the opportunities it afforded for exploring binocular vision:

Thus, many previous explorations have used stereo pictures of familiar objects or line drawings, precluding the separation of interacting cues. The investigation reported here utilized patterns devoid of all cues except binocular parallax, by using artificially created stereo images with known topological properties. Such visual displays ordinarily never occur in real-life situations, and a digital computer (with a video transducer at its output) was programmed to generate them. When these unfamiliar pictures are viewed stereoscopically, peculiar and often unexpected depth effects can be seen. (Julesz, 1960, p. 1126)

Patterns having a pseudo-random structure can be produced by hand in several ways and they have been applied to examine stereoscopic depth perception long before Julesz. The methods include distributing marks on a flat, contrasting surface; dots of either similar or different sizes have been most commonly used, but scribbles and lines have also been drawn. All the methods enlisted photography in their production. Julesz adopted a different method: The distribution of small squares was in an orderly matrix, initially an array of around 100 × 100 cells; the randomness consisted of assigning the contents of each cell in the matrix as black or white. Once a matrix was formed a region (usually a square) was displaced laterally. This was presented to one eye and the original matrix to the other. Depending on the direction of displacement the square appeared either closer to or farther from the surrounding, corresponding cells. Stereoscopic depth of the square was demonstrated independently of its monocular recognition.

## Stereoscopic Viewing

The first stereoscopes were based on mirrors, prisms or lenses (Brewster, 1849; Wheatstone, 1838, 1852) but other systems for separating the images presented to each eye were enlisted, like anaglyphs (see Blundell, 2011; Howard & Rogers, 1995, for the history of stereoscopic techniques). Anaglyphs are displays in which the left and right eye images are printed in different colours, such as red and cyan, and they are viewed through filters of the same colours. The use of colours for separating the eyes to see depth was described by Rollmann (1853); the colours that he found worked best were blue and yellow drawings combined with red and blue glasses. The general standard now adopted is for red and cyan filters for viewing similarly coloured printed or projected images and these are recommended for viewing the anaglyphs in this article (see Wade, 2021). Contemporary stereoscopic photographs of objects require to be viewed with the red filter in front of the left eye and the cyan filter for the right eye. This constraint does not apply to the stereograms in this article. In his landmark book, *Foundations of cyclopean perception*, Julesz (1971) presented stereograms both as side-by-side, black-and-white images for free viewing and as anaglyphs; anaglyph viewers were included with the book.

## Textured Stereograms Before Julesz

The novelty of Julesz's system was the use of computers to produce the RDSs. Simpler systems had been enlisted earlier, as will be discussed below in reversed chronological sequence. Revealing otherwise hidden objects in aerial stereoscopic photographs was a stimulus for Julesz to make RDSs: ‘After all, to break camouflage in aerial reconnaissance, one would view aerial images taken from two different positions (the use of parallax) through a stereoscope, and the camouflaged target would jump out in vivid depth’ (Julesz, 1991, p. 745). Claus Aschenbrenner (1954) was similarly stimulated by his work on revealing camouflaged objects from aerial stereoscopic photographs and he devised a novel means of simulating this:

A large quantity of black and white discards from a paper punch were thoroughly mixed together to assure an even mixture of distribution. The mixture was strewn on a large surface so that the density completely covered the surface. A single photograph was taken of the surface which is the background image of the stereogram. One set of letters was cut from a copy of the image, superimposed onto the background image, and photographically recorded. The letters were then displaced laterally by 1½ mm on the background photographic image and a second photographic record was produced. (The original photographs are 9 cm by 12 cm and are separated by 24 cm). (Reprinted in Shipley, 1971, p. 1491)

**Figure 1.**
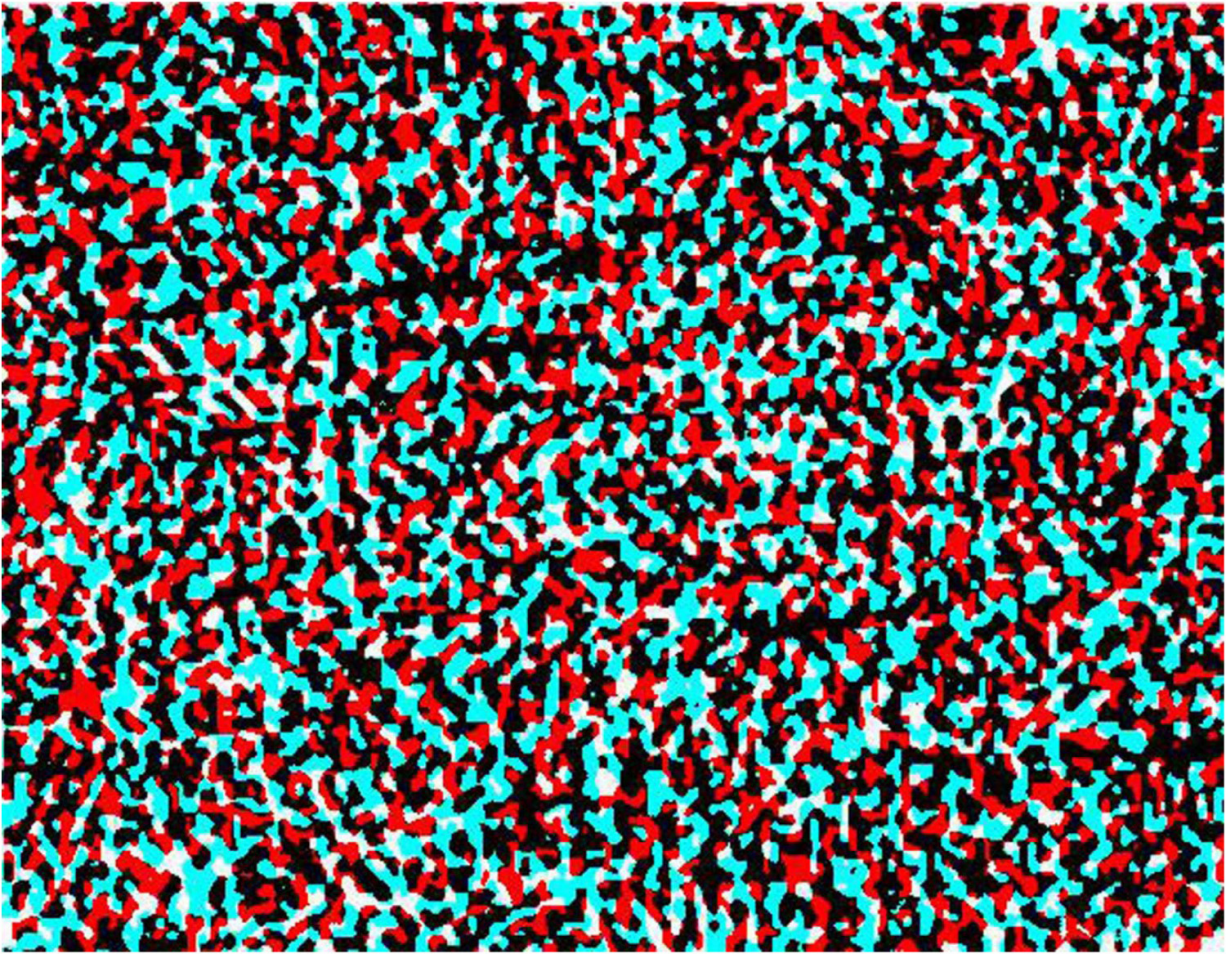
An anaglyph of Aschenbrenner's stereogram in which the word LEAK can be seen in depth. The figure is derived from an illustration of the stereo pair in Blundell (2011).

Aschenbrenner's stereogram was made to be viewed with an optical stereoscope and the side-by-side half-images are reproduced in Shipley (1971), Howard and Rogers (1995) and Blundell (2011). An anaglyph made from the stereo pair is shown in Figure 1. Aschenbrenner's method of spreading small black and white discs over a larger surface satisfies the requirements of carrier patterns for deployment in stereograms: The photographed texture was comprised of small elements of regular size irregularly distributed so that no overall distinctive features emerged. A small displacement of a region within it is difficult to detect monocularly but is the source of its appearance in stereoscopic depth.

**Figure 2.**
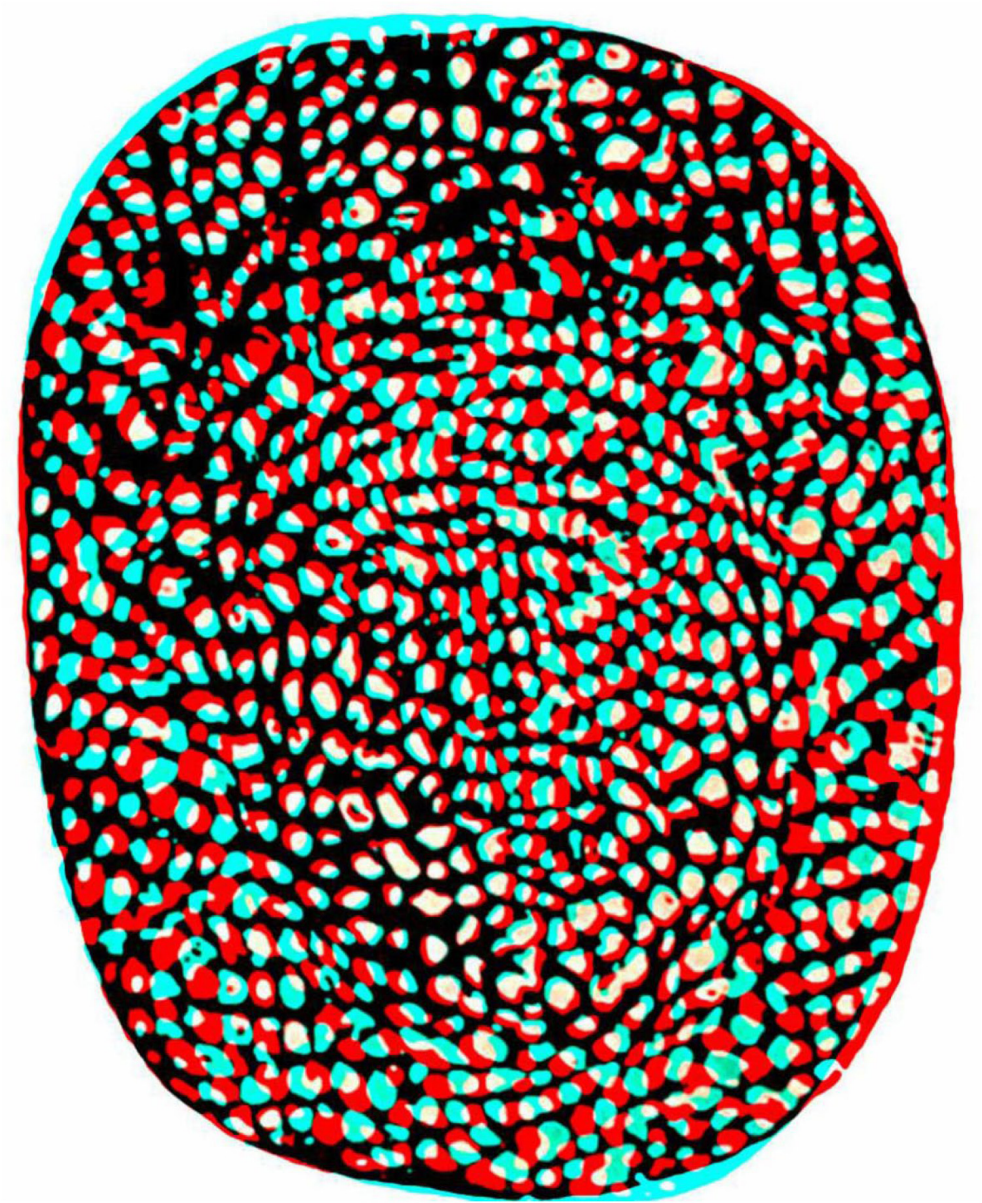
An anaglyph of Kompaneysky's stereogram in which the head of Venus was supposed to be seen in depth. The figure is derived from an illustration of the stereo pair in Howard & Rogers (1995).

Shipley (1971) referred to Aschenbrenner's construction as the first random-dot texture stereogram but others had preceded it. Paired patterns of white dots on a black background were produced by Boris Kompaneysky (1939); when viewed binocularly the face of Venus was said to appear in depth (see Howard and Rogers, 1995; Tyler, 1994). Kompaneysky's stereogram is presented as an anaglyph in Figure 2, although Kompaneysky made it for unaided viewing.

**Figure 3.**
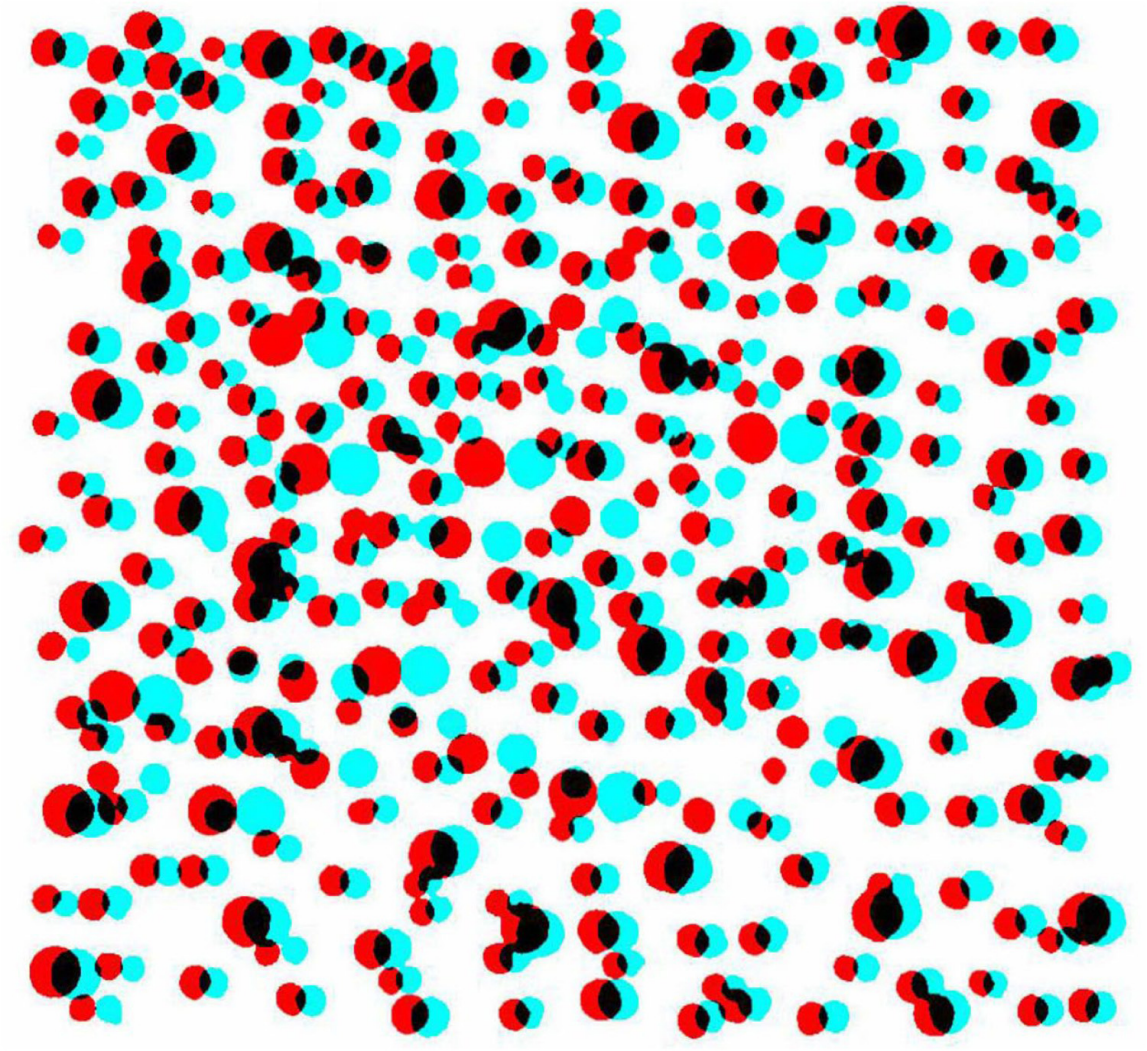
An anaglyph of Mobb’s stereogram derived from the illustration in Dennis & Patterson (1995).

Earlier still, in 1919 Herbert Mobbs, a communications engineer and keen stereoscopist, made two dot patterns in which the letter L was visible in depth when combined binocularly by over- or under-convergence. Dots of different sizes were drawn on one surface and then reproduced on a transparent surface placed over it, adding dots in the shape of the letter; the two surfaces were then photographed separately and mounted side-by-side. It had the amusing title ‘Simple Monoscopist: “Ah! The Heavens’. Sophisticated Stereoscopist: ‘No! L.”’ (Dennis & Patterson, 1995, p. 26). Dennis & Patterson refer to it as ‘almost certainly the world's first random dot stereo pair’ (p. 26). An anaglyph of Mobbs's stereogram is provided in Figure 3 for the sophisticated stereoscopist!

**Figure 4.**
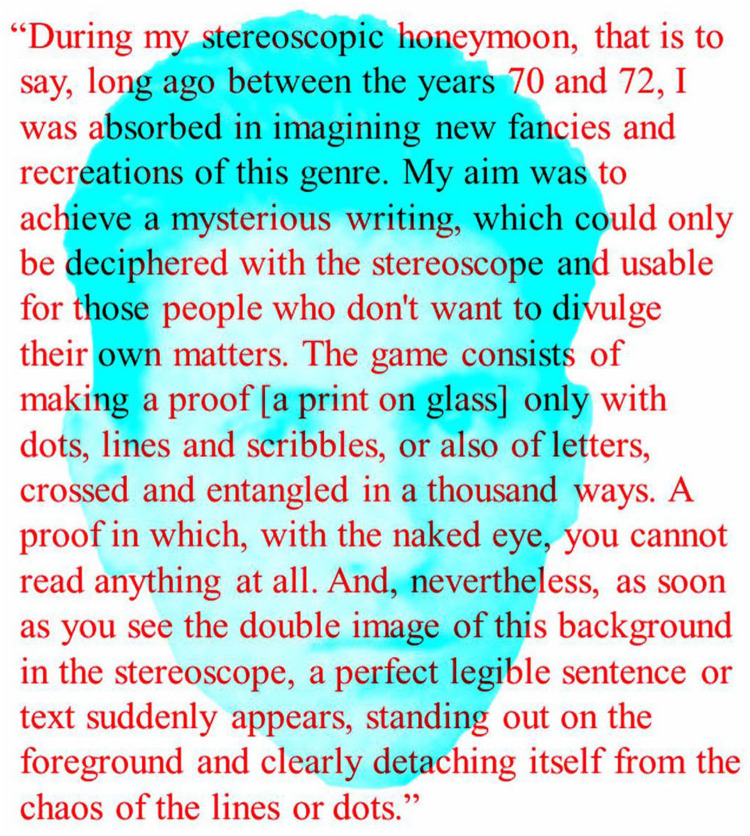
*Cajal and his description of creating stereoscopic images* by Nicholas Wade. A portrait of a youthful Cajal can be seen through the red filter and the text describing his method through the cyan filter. The text is from Bergua & Skrandies (2000, p. 71).

The use of random marks to conceal patterns monocularly and reveal them stereoscopically was devised by the microanatomist Santiago Ramón y Cajal as early as 1870, although he did not describe his technique in print until years later (Ramón y Cajal, 1901); it was translated into English by Bergua and Skrandies (2000). Cajal was a medical student at the time and he had developed a fascination with stereophotography as a means of concealment. His portrait is shown in Figure 4, together with the text describing his method.

**Figure 5.**
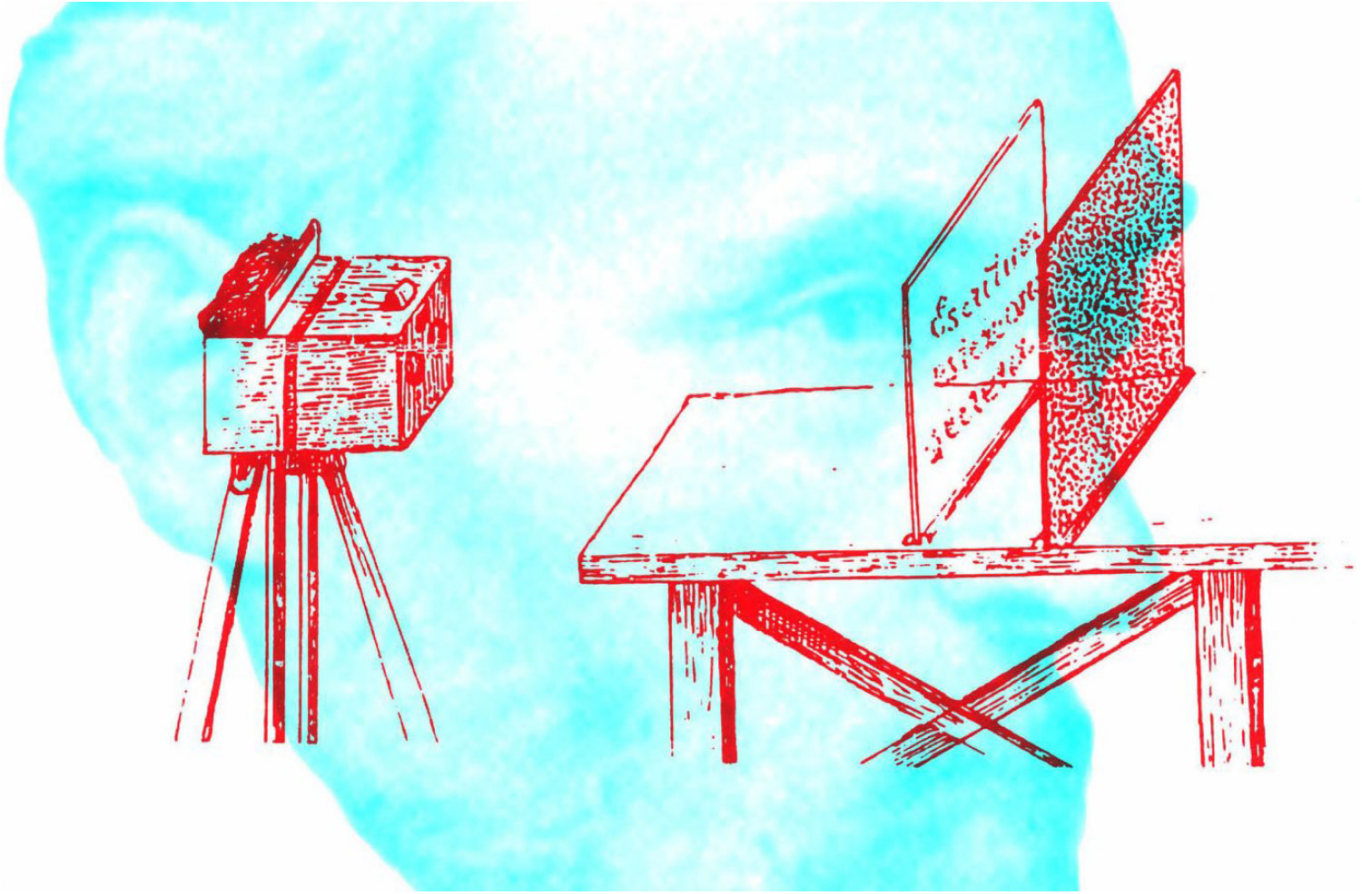
*Cajal and his arrangement for concealing messages stereoscopically* by Nicholas Wade. A portrait of a Cajal in middle age can be seen through the red filter and his arrangement for taking stereoscopic photographs through the cyan filter.

The separated patterns on glass and board were photographed with a binocular camera and the two half-images were viewed in a stereoscope. Cajal's diagram of the arrangement is shown in Figure 5 together with a portrait from around the time his description of it was published.

**Figure 6.**
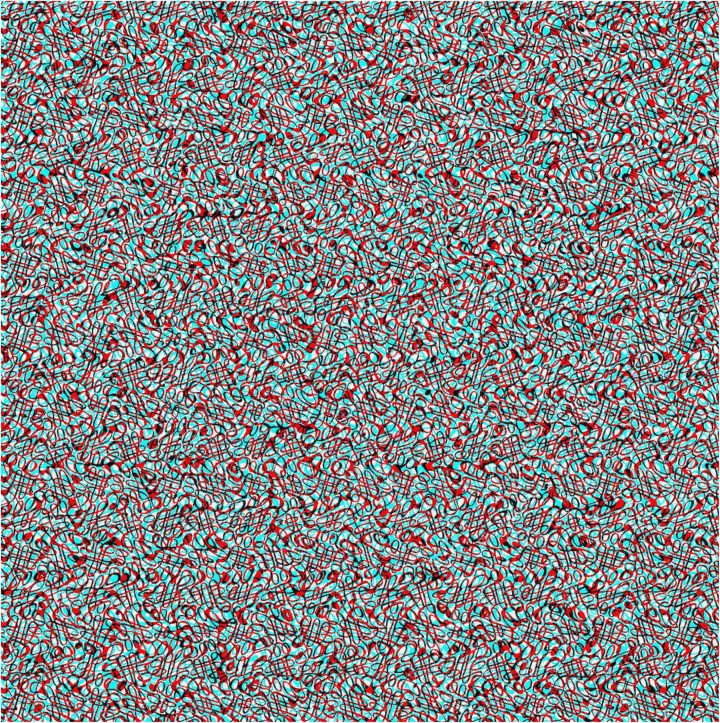
An anaglyph in which a disc is seen in depth. The two half-images are displaced by equal amounts left and right as would be the case for binocular photographs taken using Cajal's method.

It is instructive to compare the methods of Cajal and Julesz for producing stereoscopic depth from two patterns. By using a binocular camera and viewing the photographs in a stereoscope, Cajal created disparities in both half-images relative to their common backgrounds. An anaglyph analogous to this is illustrated in Figure 6. The carrier pattern is made up of dense squiggles not unlike those described by Cajal.

**Figure 7.**
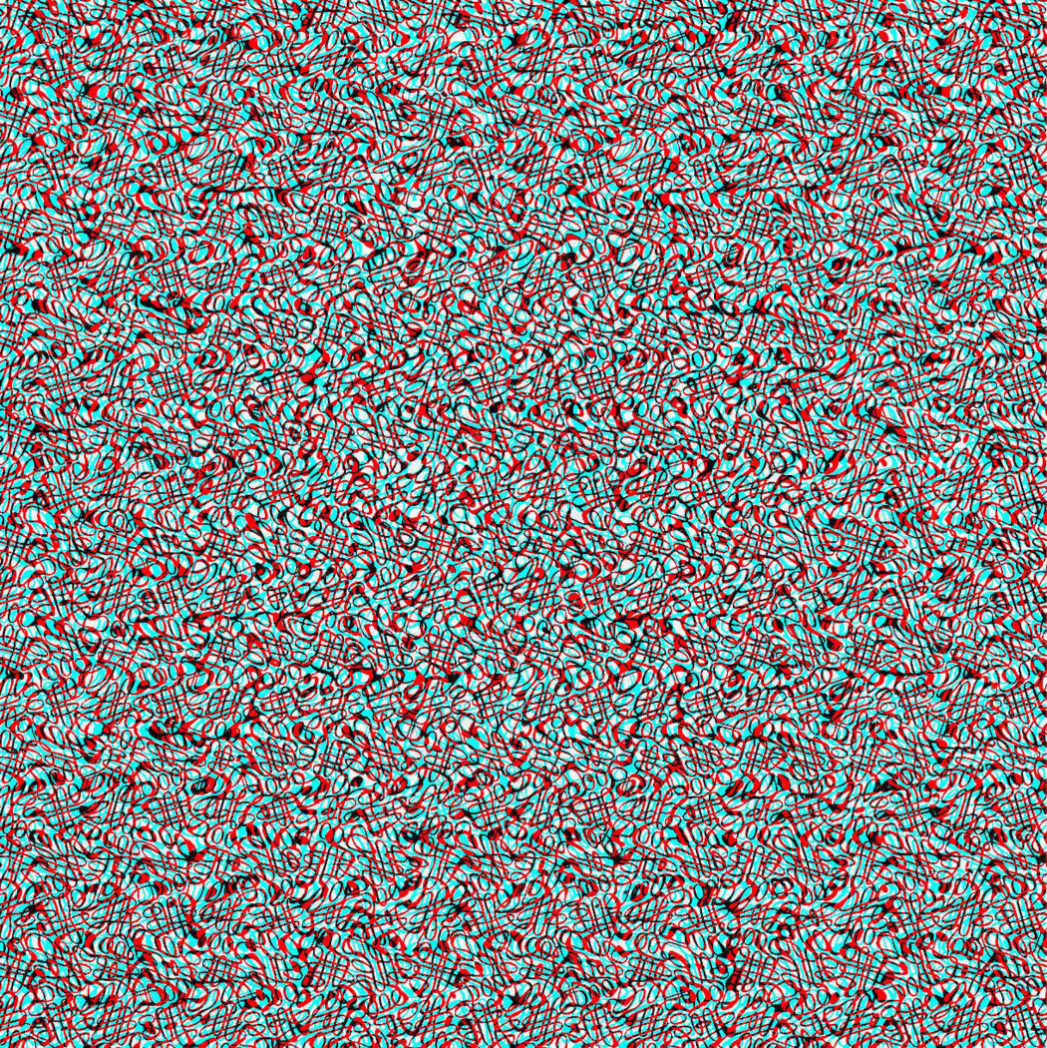
An anaglyph in which a disc is displaced in one half-image only. The total displacement is the same as that in Figure 6.

The method Julesz adopted for creating RDSs involved displacing a region in one half-image only, as illustrated in Figure 7. The total disparity is the same as that with the Cajal method (Figure 6) and the apparent depth appears the same.

Thus, it would seem that Cajal produced stereograms very similar to Julesz's RDSs. The major difference between them is that Julesz appreciated their significance whereas Cajal did not. Cajal was more interested in the crypotographic possibilities offered by such concealment:

This singular cryptographic correspondence is a bit annoying, however, on the other hand, it is one of the safest known. It does not matter if one of the proofs falls accidentally into the hands of someone curious, as for the deciphering, we need both proofs and their adequate matching. For better security it is convenient to send both proofs in a consecutive way, that is to say, the second one will be sent not without prior notice of the receipt of the first one. (Bergua & Skrandies, 2000, p. 72).

Cajal was disparaging about his own discovery: ‘My little invention is, in fact, a puerile game unworthy of publishing, but it really amused me at that time’ (Bergua & Skrandies, 2000, p. 71). In fact, it preceded the RDSs of Julesz by almost 90 years.

Bergua & Skrandies suggest that Mach (1866) ‘constructed stereoscopic stimuli that contained no visible contours’ (p. 70). Mach does have a section of the article devoted to stereoscopy and pseudoscopy but there is no mention of any stimulus resembling those described above. The stimulus that contained no visible contours is what is now known as ‘Mach's book’ and it is not stereoscopic; it is a simple diagram of two touching and symmetrical parallelograms which can reverse in apparent depth. In fact, the depth reversals are easier to see with one eye than with two. There is no mention of patterned stereograms either in this article or in the consideration of stereoscopy in his *Analysis of sensations* (Mach, 1886).

**Figure 8.**
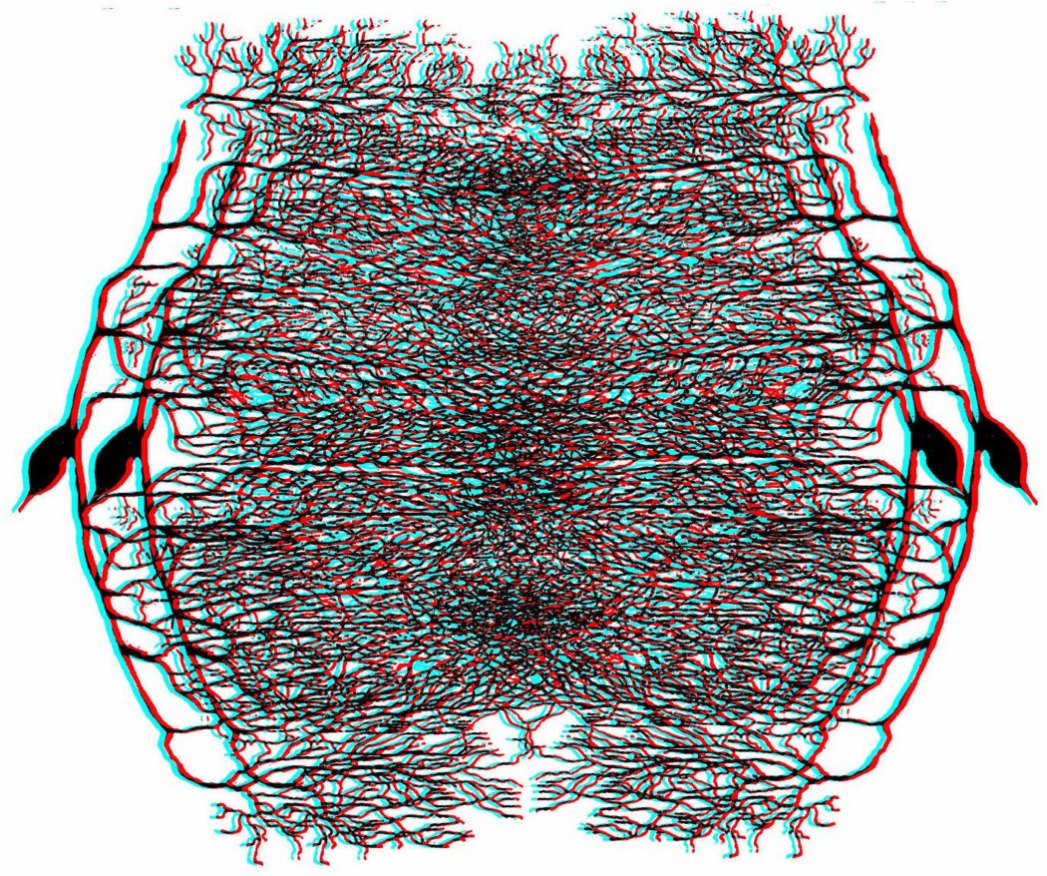
*Cajal’s Purkinje cells* by Nicholas Wade. An anaglyph in which a central disc appears in stereoscopic depth. The carrier pattern is derived from Cajal's representation of a Purkinje cell and its arborizations as seen when using Golgi's staining method.

The range of patterns that can be used to construct the perceptual equivalents of RDSs is immense (see Wade, 2021, 2023a, 2023b, 2024) and an unusual one is employed in Figure 8. The pattern is derived from a drawing of a Purkinje cell made by Cajal in 1899, around the time he published his account of stereoscopic cryptography; the arborizations make a dense texture when overlaid and a central disc can be seen in stereoscopic depth.

## Why Didn’t Wheatstone Make Textured Stereograms?

**Figure 9.**
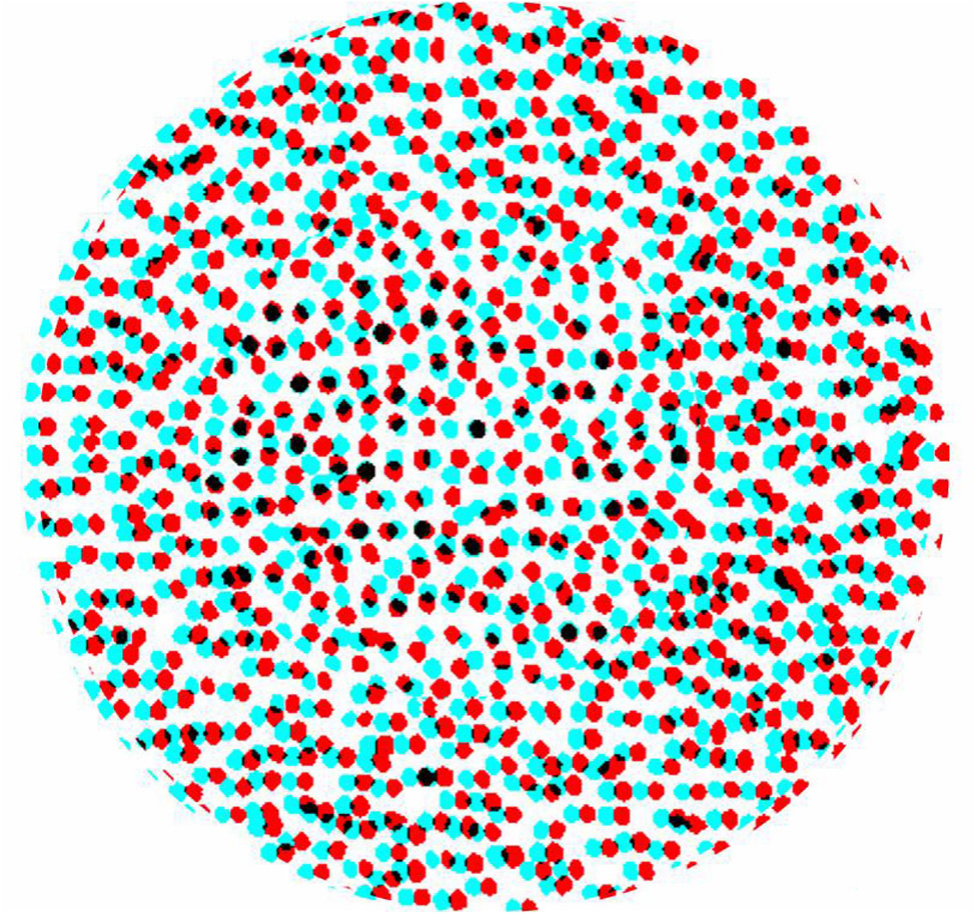
*Textured dot anaglyph* by Nicholas Wade. The carrier pattern is comprised of hand-drawn irregular dots, as can be seen through the cyan filter and the same pattern with a displaced disc through the red filter. With binocular viewing the disc appears more distant with the red filter in front of the left eye and the cyan filter for the right eye. The apparent depth is reversed when the eye/filter arrangement is reversed.

Wheatstone was an expert on cryptography. He even deciphered messages in letters to the *Times* and in one case, published an answer using the same cipher system (Bowers, 1975). More importantly, Wheatstone devised instruments to assist in encrypting messages sent by telegraph. Independently of this, Wheatstone could have made textured stereograms because computers are not essential for their production. He could have drawn an irregular pattern of dots, cut out and displaced a region laterally and paired it in a stereoscope with the original pattern. Figure 9 is an anaglyph made from a hand-drawn dot pattern; the dots are clearly neither regular nor random but they provide a simple textured carrier pattern so that a region (a disc) can be displaced laterally without appearing different from the original.

**Figure 10.**
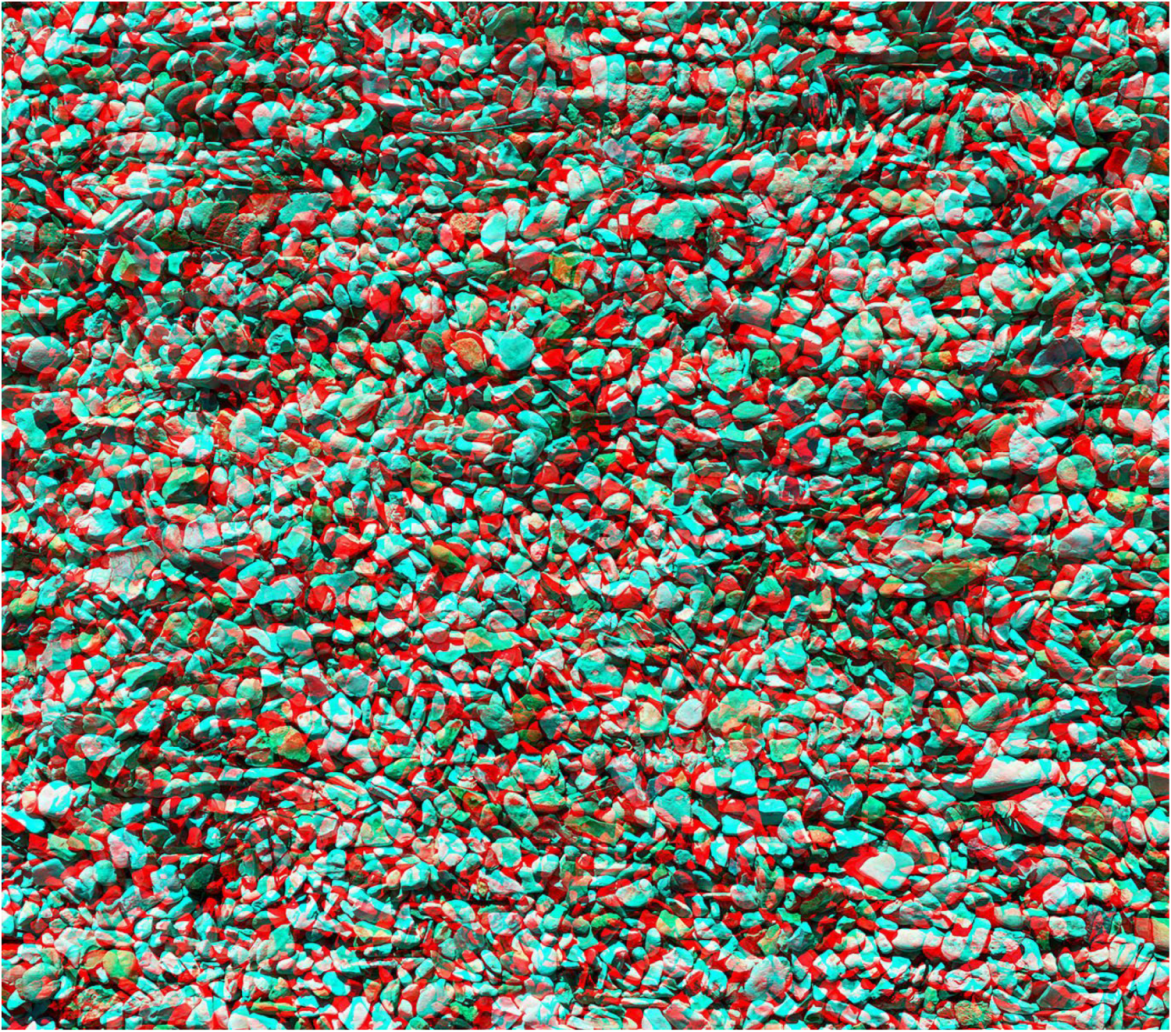
*Stone square* by Nicholas Wade.

Wheatstone was a keen photographer and a friend of Henry Talbot, who invented paper-negative photographs (see Pellerin & May, 2021). Moreover, Wheatstone was an advocate of the assistance photography could offer to stereoscopy. In his second memoir on stereoscopic vision, he wrote: ‘ What the hand of the artist was unable to accomplish, the chemical action of light, directed by the camera, has enabled us to effect’ (Wheatstone, 1852, p. 7). Accordingly, it would not have been difficult for him to photograph a textured surface, print the negative twice to make two positives, displace a region in one of them and view them in a stereoscope. Figure 10 provides an example of how this can be achieved; a photograph of stones is presented to one eye and the same pattern with a square displaced laterally to the other.

This should not be taken as a criticism of Wheatstone. The same argument has been made for the stereoscope itself (Wade, 1987) and Wheatstone invented that instrument. The first stereoscopes were constructed from mirrors, lenses and prisms and these had been available for many centuries. The critical feature was appreciating the problem that a stereoscope could address. It was not invented earlier because there was no accepted phenomenon that the instrument could examine. Wheatstone's invention resulted in stereoscopic depth perception becoming an experimentally tractable feature of binocular vision.

## Wheatstone's Stereogram Without Monocular Recognition

Wheatstone did realise the appropriate question in the case of stereograms. How can stereoscopic depth perception be studied independently of monocular cues in the stimuli? Most of the stimuli he devised were outline drawings of geometrical objects but one was comprised of five horizontally separated dots in each eye with the separations between the dots greater in one pattern (Wheatstone, 1838, Figure 11). The stereogram does satisfy the requirement of having no indication of the stereoscopic depth available to either eye alone but depth can be seen binocularly.

**Figure 11.**
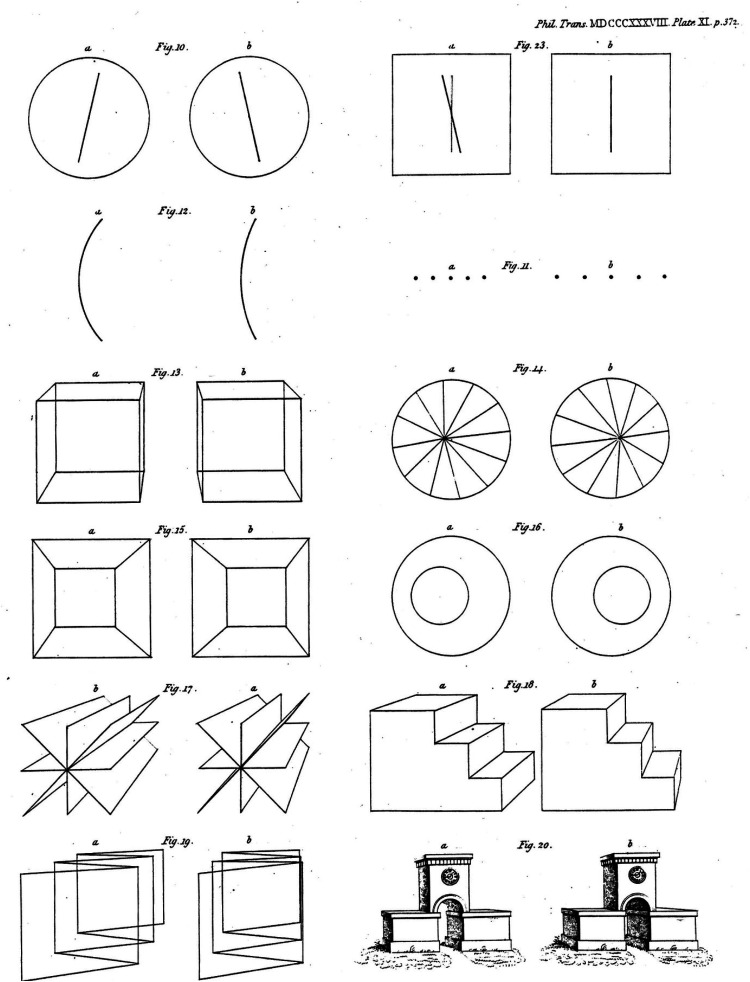
Stereograms from Wheatstone (1838).

Before presenting an anaglyph of Wheatstone's stereogram, something needs to be said about those printed in his article, which are shown in Figure 11. The first point is that the half-images are presented as they would have been mounted on the boards of Wheatstone's mirror stereoscope, not as they would have been reversed by reflection. This was so despite Wheatstone's statement that:

As the drawings are reversed by reflection in the mirrors, I will suppose these figures to be the reflected images to which the eyes are directed in the apparatus; those marked *b* being seen by the right eye, and those marked *a* by the left eye. The drawings, it has been already explained, are two different projections of the same object seen from two points of sight. (Wheatstone, 1838, p. 376)

**Figure 12.**
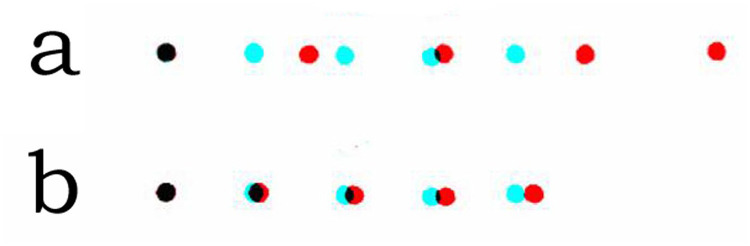
Anaglyphs of a line of five dots with different separations in each eye. (a) After Figure 11 in Wheatstone (1838) and (b) modified so that the separation difference between the left and right eye stimuli is reduced to 5%. In (b) the monocular images are indistinguishable but the line of dots appears to recede toward the right with the red filter in front of the left eye and to approach with the red filter in front of the right eye.

**Figure 13.**
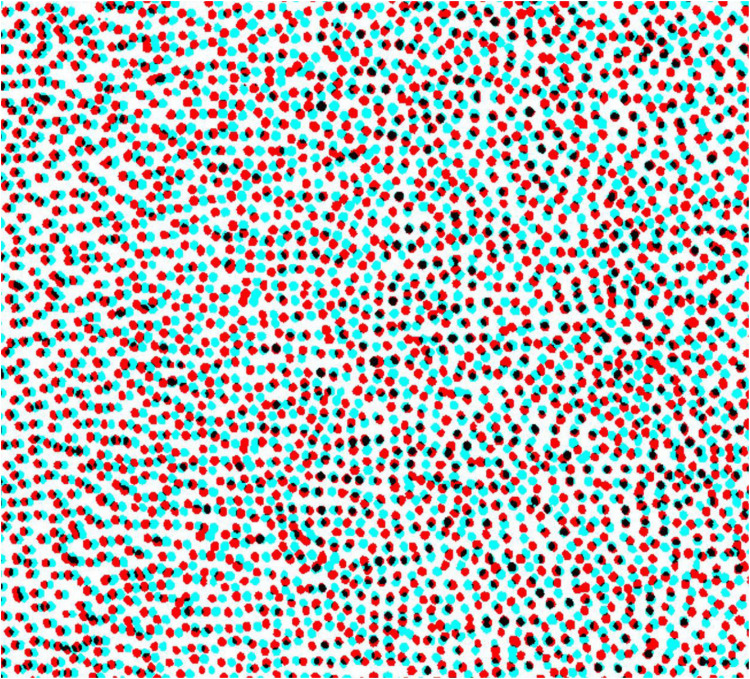
*A surface in stereoscopic slant* by Nicholas Wade. Viewing the pattern through the red filter with the left eye and the cyan filter with the right results in the right side appearing closer than the left; the reverse occurs when the filter/eye arrangement is reversed.

For whatever reason, this is not the case for the stereograms printed in Wheatstone's article. Take the perspective representations of a cube (his Figure 13); *a* is clearly a right-eye view and *b* the projection to the left eye. Moreover, despite stating that the drawings are different projections of the same objects, it is not evident what the objects represented by his Figures 10–12 are.

The second point is that disparities between the half-images are excessive in Wheatstone's printed diagrams. There is little in the descriptions Wheatstone gives about perception of the stereograms that the ones printed are the same as those he used for his experiments. No difficulties in combining the half-images are mentioned. For example, the angular separation of the inclined lines in his Figure 10 is approximately 25°, far beyond the fusional range possible. The exaggeration is even more pronounced in his Figure 11, with five dots aligned horizontally: The separations in the right eye image are around 60% greater than those in the left and the two half-images cannot be combined, as shown here in Figure 12a. When the separations are reduced, as in Figure 12b, the effect described by Wheatstone can be seen: ‘A series of points all in the same horizontal plane, but each towards the right hand side successively nearer the observer’ (1838, p. 376). As would be expected from the first point above, the direction of depth seen in Figure 12b is the opposite of Wheatstone's account. It is probable from his descriptions the stimulus he viewed was more similar to Figure 12b rather than 12a. If that is so, it can be said that Wheatstone produced the first stereogram in which the depth experienced cannot be seen in either monocular image. However, Wheatstone did not make this claim himself; his main reason for presenting the stereogram was to demonstrate that monocular stimuli of different separations can be combined. It was an element in Wheatstone's evidence against the doctrine of identical retinal points defining binocular single vision. In addition, his Figure 23 was designed to show that stimulation of identical retinal points could lead to diplopia (see Ono & Wade, 1985).

## Conclusion

The desire to produce stereoscopic pictures in which an enclosed region appeared in depth, despite the absence of monocular depth cues, was voiced by Wheatstone but was not realised until textured stereograms were produced. Several were introduced before Julesz constructed RDSs using a computer. Perhaps the earliest was Cajal's method for creating stereoscopic images devoid of monocular recognition. His method was similar in principle to that adopted by Julesz and the stereoscopic outcomes are equivalent. Both methods resulted in shapes rather than objects appearing in depth. Computers are not necessary for creating random-dot-like patterns but Wheatstone did not make this step. Similarly, photographing textures and displacing regions within them was a technique available to him. Perhaps he did not use these methods because he was concerned with representing stereoscopic objects rather than surface shapes. As Julesz observed: ‘Such visual displays ordinarily never occur in real-life situations’ (1960, p. 1126). The patterns need to be dense in texture like the dots and scribbles used prior to Julesz.

Wheatstone (1838) did produce a stereogram comprised of a line of dots with different separations in each eye that appeared slanted in depth; there was no recognition by either eye alone of the depth that could be seen when the patterns were viewed in a stereoscope. He did not recognise the significance of the stereogram possibly because he was more concerned with representing objects rather than lines or surfaces stereoscopically. Had the dots been multiplied and scrambled spatially then a surface in depth, rather than a line, would have been seen, as in Figure 13.
